# Genetic variations of E6 and long control region of human papillomavirus type 16 from patients with cervical lesion in Liaoning, China

**DOI:** 10.1186/1471-2407-13-459

**Published:** 2013-10-07

**Authors:** Zhengrong Sun, Zhitao Lu, Jianhua Liu, Guili Wang, Weiqiang Zhou, Lianxia Yang, Chao Liu, Bo Wang, Qiang Ruan

**Affiliations:** 1 Present address: Virus Laboratory, The Affiliated Shengjing Hospital, China Medical University, No 36, Sanhao Street, Heping District, Shenyang 110004, China; 2 Department of clinical laboratory,The Affiliated Shengjing Hospital, China Medical University, Heping District, Shenyang 110004, China

**Keywords:** HPV16, E6, LCR, Cervical lesion

## Abstract

**Background:**

High-risk human papillomavirus type 16 (HPV16) is a risk factor for cervical cancer. Previous studies suggest that polymorphisms in the E6 gene or the long control region(LCR)of HPV16 may alter the oncogenic potential of the virus. The aims of this study were to investigate the genetic variations of HPV16 E6 gene and LCR in isolates from Chinese population and correlation of the E6 and LCR polymorphisms with disease status of infected patients.

**Methods:**

HPV16 positive endocervical specimens were collected from 304 women living in Northeast of China. Sequences of E6 gene and LCR were analyzed by PCR-sequencing.

**Results:**

Two lineages were found in the populations, including EUR lineage and As lineage. Based on the HPV16 prototype, the most frequent variation in the E6 gene was T178A/G (48.7%), followed by mutations of G94A (12.2%) and T350G (9.9%). The rank orders of incidence of E6 variations in amino acid were as follows: D25E (46.3%), L83V (9.9%) and H78Y (4.3%). Nucleotide variations in LCR were found in all the 304 isolates from HPV16 positive cervical samples. The most commonly observed LCR variations were the transition replacement G7193T, 7434CIns, G7521A and 7863ADel (100%). The As lineage was associated with HPV persistent infections and with disease status of ≥CIN2,3. The EUR lineage variants showed a negative trend of association with the severity of ≥CIN2,3. Among 41 variations found in LCR, 25 (61.0%) were located at the binding sites for transcription factors. Occurrence of ≥CIN2,3 was significantly associated with the mutations of R10G/L83V in E6 and the C7294T co-variation in LCR, after adjusting for ages of infected patients.

**Conclusions:**

Associations between As lineage and HPV persistent infections, and with disease status of ≥CIN2,3, and an association between the EUR lineage and negative trend of association with the severity of ≥CIN2,3 were found in this study. An association between a co-variation of R10G/L83V in E6 and C7294T in LCR and an increased risk for developing CIN-2,3 was found in a HPV16 infected population of Chinese women. These findings indicate that HPV16 polymorphism influences development of CIN-2,3.

## Background

Persistent infections of high-risk human papillomavirus (HPV) are the necessary causes of cervical cancer. However, only a minority of women infected with high-risk HPV will develop persistent infection [1]. Developing risk of cervical intraepithelial neoplasia grade 2 or 3 (CIN2,3) is affected by viral, environmental and host factors, which are also thought to play a mediating role in cervical carcinogenesis [2]. HPV has coevolved through time with humans into distinct genotypes [3,4]. In a given type, the HPV genome is further classified into variants through cumulative nucleotide variations less than a 5% difference. Epidemiological evidence suggests that polymorphism of HPV genome could contribute to viral pathogenicity and oncogenicity [4,5]. A better understanding of the association between HPV polymorphism, persistent infection and risk for developing cervical cancer could help explain why only a subset of women with HPV infection develop cervical cancer.

HPV type 16 (HPV16) is the most prevalent high-risk (HR) type worldwide and is found in the majority of cervical cancer cases [6,7]. The HPV16 genome is about 7,900 bp long and consists of 8 protein-coding genes (L1, L2, E1, E2, E4, E5, E6, and E7) and 2 non-coding regions, including the non-coding region (NCR) and the long control region (LCR) [8]. Proteins encoded by E6 and E7 genes are the major oncoproteins, which are highly expressed in tumors and are involved in tumorgenesis. LCR, adjacent to E6 downstream, contains the early promoter and regulatory elements involved in viral DNA replication and transcription.

Based principally on LCR sequences, several studies from Europe and American have suggested that HPV16 variants can influence viral persistence and development of cervical cancer [9-11]. The aims of this study were to detect polymorphism of HPV16 E6 gene and LCR in isolates from women without a history of immunosuppression in northeast China, and to investigate the association of polymorphisms of HPV16 E6 gene and LCR with persistent HPV16 infection and developing risks of CIN2,3 or cervical cancer.

## Methods

### Study population

Three hundred and four HPV16 positive cervical cytobrush samples detected with flow-through hybridization (Hybribio Ltd, Hong Kong) were collected from women admitted to the Affiliated Shengjing hospital of China medical university. According to the cytological and histological evaluations of fresh specimens, cervical disease statuses of the women were categorized as normal, cervicitis, atypical squamous cells of undetermined significance (ASCUS), cervical intraepithelial neoplasia grade 1, 2 and 3 (CIN1, CIN2 and CIN3) and cervical cancer (CC). Histopathological diagnosis was completed by a pathologist who was unaware of the HPV detection results. Informed consent was obtained from participation in the study and this study was approved by the ethics committee of the Affiliated Shengjing hospital.

### Analysis of HPV16 E6 and LCR by PCR-sequencing

To obtain HPV16 E6 and LCR sequences, a half-nested PCR was performed as that previously described [12]. According to the reference strain (GenBank KO 2718), the primers used in the amplification were designed as follows: the forward primer of LCR-F (5’-ACGCAAAAAACGTAAGCTG-3’) is located at nucleotide (nt) 7133–7151, the out backward primer of E6-R (5’-CTTCCCCATTGGTACC TGCAG-3’) at nt 875–895 and the inner backward primer of E6-R-2 (5’-TCCATGCATGATTACAGCTGGGTT-3’) at nt 547–570. In the first-round PCR, 10–100 ng of sample DNA was used as a template; in the second-round reaction, 1μl of the first-round products were used. PCR was performed in a 9700 Thermal Cycler (Perkin–Elmer GeneAmp PCR system) with the following parameters for all the amplification reactions: an initial denaturation at 94°C for 5 min, followed by 30 cycles of 94°C for 45 sec, 50°C for 45 sec and 72°C for 1 min, and a final elongation at 72°C for 5 min. A negative control reaction without DNA template was included in all PCR tests.

The size of the PCR products containing HPV16 E6–LCR sequence was confirmed by gel electrophoresis. The amplicons were purified using the QIAquick gel extraction kit (Qiagen Inc, Mississauga, ON). Direct double-stranded PCR sequencing was performed for the E6-LCR amplicons using the same primers as in PCR by fluorescent cycle-sequencing method (BigDye terminator ready reaction kit; Perkin-Elmer). All amplicons were sequenced at least twice to exclude Taq-induced errors. Sequences of the amplicons were aligned by CLUSTAL W (version 1.8). The obtained sequences were compared to the reference sequence (GenBank KO 2718) using NCBI BLAST. The HPV16 sequences were classified into respective variant classes as: prototype (GenBank KO 2718), European (E), Asian (As), African 1 (Af-1), African 2 (Af-2) and Asian American (AA) [13-15]. To assess the effects of LCR variations on binding sites for cellular transcription factors, the TFSEARCH software was used [16].

### Data analysis

The association of HPV16 polymorphisms with persistent infection and developing risks of CIN2,3 or cervical cancer of women was investigated. Persistent infection was conceptualized at least two times positive for HPV detection in a series of samples collected at more than three visits. The proportion of women infected with HPV16 prototype was compared between participants with transient and persistent infection by Fisher’s exact test. In this study, CIN1 or low-grade squamous intraepithelial lesion (LSIL) is not considered as a precursor lesion to cervical cancer but a morphologic consequence of HPV infection. So, the controls including women without CIN or with CIN1 and LSIL are designated as <CIN2,3. After classifying the isolates into variant lineages, the association of HPV16 polymorphisms in women with CIN2,3 or cancer (≥CIN2,3) was analyzed. The rate of HPV16 variants in 167 women with ≥CIN2,3 were compared to that in 137 women with <CIN2,3. The magnitude of the associations of HPV16 variants with ≥CIN2,3 was assessed by calculating odds ratios (OR) and respective 95% confidence intervals (CI). The association of HPV16 polymorphism in isolates from women with ≥CIN2,3 was assessed individually for HPV16 each variations. Fisher’s exact test was used to assess the significance of the differences between dichotomous variables, and a Mann–Whitney test was used to assess the significance of the differences of age between groups. Unconditional multiple logistic regression analysis was performed to obtain maximum likelihood estimates of the OR while controlling for the confounding effects of study site, age and smoking. Statistical analyses were performed with STATISTICA version 6 Software (StatSoft, Tulsa, OK).

## Results

Information about the studied population is shown in Table 1. The grade of cervical disease of the 304 participants were 95 without squamous intraepithelial lesion(SIL), 10 low-grade squamous intraepithelial lesion (LSIL) diagnosed by cytology, and 32 CIN1, 117 CIN2,3 and 50 cancers diagnosed by histopathology. The median age of the participants was 46 years. Among the populations, 121 women had never smoked tobacco, 124 had ever smoked (35 were current smokers) and 59 had unknown smoking habits.

**Table 1:** Information about HPV16 infected women

**HPV 16-posotive women (%) n=304**	
**Age (years)**	
Mean ± SD	48.9 ± 11.7
Median	46
**Smoking (no. of women)**	
Never	121 (39.8)
Ever	124 (40.8)
Unknown	59 (19.4)
**Cervical disease (no. of women)**	
Normal	95 (31.3)
LSIL	10 (3.3)
CIN1	32 (10.5)
CIN2,3	117 (38.5)
Cancer	50 (16.4)
**Persistence (no. of women)**	
Persistence	168 (55.3)
Transient	106 (34.9)
Undefined	30 (9.9)
**Persistence in HPV 16 European T350G (no. of women)**	
Persistence	16 (9.5)
Transient	12 (11.3)
Undefined	2 (6.6)
**Persistence in As lineage (no. of women)**	
Persistence	81 (48.2)
Transient	22 (20.6)
Undefined	10 (33.3)

### Genomic polymorphisms of HPV16 E6 and LCR

Tables 2 and 3 display the sites and natures of the variations found in E6 and LCR in a total of 304 HPV16 positive cervical samples, and the changes in amino acid sequences of E6 proteins compared to that of the HPV16 prototype. The results showed that the predominant variant among women infected with HPV16 was the EUR lineage (62.8%, 191/304), followed by As (37.2%, 113/304). Among the EUR lineage, the variants E350G and E131G were seen in 9.9% (30/304) and 3.0% (9/304) of the 304 women, respectively. Several variations in E6 and LCR were co-segregated, including T178G in E6 gene and T7201C, C7270T, A7287C, A7289C, A7730C, G7842A in LCR, which were observed in 106 isolates (34.9%, 106/304).

**Table 2:** Variations of HPV 16 E6 gene in strains from patients with different grades of cervical lesions

**Variation of HPV-16 E6 at nucleotide position (450bp)**										**Grade of Related Cervical lesion**				
**Categories**	**94**	**131**	**176**	**178**	**215**	**241**	**335**	**350**	**442**	**Normal n =95**	**CIN1/LSIL n =42**	**CIN2-3 n =117**	**CC n =50**	**Total n=304**
**KO2718**	**G**	**A**	**G**	**T**	**C**	**T**	**C**	**T**	**A**					
**Variation**	**A**	**C/G**	**A**	**A/G**	**T**	**G**	**T**	**G**	**C**					
EUR	_	_	_	_	_	_	_	_	_	10	5	4	5	24
EUR	a	_	_	_	_	_	_	_	_	10	2	18	0	30
EUR	_	_	_	_	t	_	_	_	_	10	2	0	0	12
EUR	_	_	_	_	_	g	_	_	_	6	10	2	4	22
EUR	_	_	a	_	_	_	_	_	_	12	4	1	0	17
EUR	_	_	_	_	_	_	_	_	C	5	0	0	0	5
EUR	_	_	_	_	_	_	T	_	_	11	2	0	0	13
EUR	_	_	_	A	_	_	_	_	_	7	4	17	10	38
EUR	a	_	_	_	_	_	_	G	_	4	0	0	0	4
EUR	a	_	_	_	_	_	_	G	C	3	0	0	0	3
EUR	_	G	_	_	_	_	_	G	_	0	1	8	0	9
EUR	_	_	_	_	_	_	_	G	_	3	2	9	0	14
As	_	c	_	G	_	_	_	_	_	6	3	4	19	32
As	_	_	_	G	_	_	_	_	_	8	7	54	12	81
AminoAcid substitution	_	R10G	_	D25E	_	_	H78Y	L83V	E113D					

Note: HPV 16 prototype (GenBank accession number KO 2708) was used as the reference. Nucleotide positions in E6 are presented at the top of the table according to the reference sequence. Amino acid substitutions are indicated under the corresponding amino acidic position. Nucleotide changes are shown by the corresponding letters. Dashes indicate positions at which no variation was found. Capital letter indicates synonymous mutation; Lower-case character indicates non-synonymous mutation. E-p,Europen prototype; CC, cervical cancer.

**Table 3:** Variations of HPV 16 LCR in strains from patients with different grades of cervical lesions

**Nucleotide variations**	**Transcription factors**	**No. of women with cervical different grades of cervical lesions dysplasia**				
		**Normal n=95**	**CIN1/LSIL n=42**	**CIN2–3 n=117**	**CC n=50**	**Total n=304**
^**N**^**G7193T**	**SRY**	**95**	**42**	**117**	**50**	**304**
**T7201C**	**SRY**	**65**	**8**	**17**	**16**	**106**
**T7228A**	**CdxA**	**17**	**4**	**0**	**8**	**29**
**A7238C**	**CdxA**	**25**	**4**	**8**	**0**	**37**
**T7238A**	**RAP1**	**17**	**4**	**0**	**8**	**29**
**C7270T**	**USF**	**65**	**8**	**17**	**16**	**106**
**A7279T**	**USF**	**0**	**5**	**8**	**4**	**17**
**A7287C**		**65**	**8**	**17**	**16**	**106**
**A7289C**		**51**	**8**	**13**	**8**	**80**
^**N**^**C7294T**	**CdxA**	**0**	**4**	**8**	**10**	**22**
^**N**^**G7319A**		**0**	**4**	**8**	**10**	**22**
^**N**^**A7326G**		**0**	**4**	**8**	**10**	**22**
**A7339T**	**SRY**	**12**	**4**	**0**	**8**	**24**
^**N**^**T7350A**	**Dfd, S8**	**0**	**4**	**8**	**10**	**22**
^**N**^**T7368G**	**CF2-II**	**0**	**4**	**8**	**10**	**22**
^**N**^**7434C(Ins)**		**95**	**42**	**117**	**50**	**304**
**C7436T**	**E2**	**95**	**42**	**117**	**50**	**304**
**G7447T**		**12**	**4**	**0**	**8**	**24**
**G7456A**		**12**	**4**	**0**	**8**	**24**
**G7502T**	**CdxA**	**17**	**4**	**4**	**8**	**33**
^**N**^**T7503G**	**HSF**	**0**	**4**	**8**	**10**	**22**
^**N**^**A7515C**	**AP-1,cap**	**0**	**4**	**8**	**10**	**22**
**G7521A**		**95**	**42**	**117**	**50**	**304**
^**N**^**G7553T**	**Sox-5, SRY,HFH-2**	**0**	**4**	**8**	**10**	**22**
^**N**^**A7602G**	**P**	**0**	**4**	**8**	**10**	**22**
**T7608C**	**Hb**	**8**	**0**	**4**	**4**	**16**
**G7677A**	**E2F**	**12**	**4**	**0**	**8**	**24**
**G7683A**	**E2F**	**12**	**4**	**0**	**8**	**24**
**T7714G**		**19**	**7**	**2**	**2**	**30**
**A7730C**	**Oct-1**	**65**	**8**	**17**	**16**	**106**
^**N**^**A7740T**	**Oct-1**	**0**	**4**	**8**	**10**	**22**
**T7755A**		**12**	**4**	**0**	**8**	**24**
**T7781C**	**cap**	**25**	**0**	**17**	**0**	**42**
**T7841A**		**12**	**4**	**0**	**0**	**16**
**G7842A**		**65**	**8**	**17**	**16**	**106**
^**N**^**7863A(Del)**		**95**	**42**	**117**	**50**	**304**
**G7868A**		**28**	**0**	**9**	**4**	**34**
**T7880C**	**CdxA**	**12**	**4**	**0**	**8**	**24**
**A7884C**		**12**	**4**	**0**	**8**	**24**
**G7885A**		**12**	**4**	**0**	**8**	**24**
**C7930T**	**SRY**	**65**	**8**	**17**	**16**	**106**

Note: HPV 16 prototype (GenBank accession number KO 2718) was used as the reference. Nucleotide positions in LCR are presented according to the reference sequence. Novel variations are labeled as 'N’.

Nucleotide variations of HPV16 E6 were found in 92.1% (280 of 304) of the HPV16 positive cervical samples, whereas amino acid variations were found in 65.5% (199 of 304). Nucleotide variations of E6 included 4 silent mutations of G94A, G176A, C215T and T241G and 5 missense mutations of A131C, T178G/A, C335T, T350G and A442C, which led to amino acid variations of R10G, D25E, H78Y, L83V and E113D, respectively. The most frequent nucleotide variation was T178G/A (49.7%, 151 of 304), in which T178G was found in 113 strains and T178A in 38 strains (Table 2). The rank orders of E6 variants in amino acid were as follows: D25E (49.7%, 151/304), L83V (9.9%, 30/304), H78Y (4.3%, 13/304), R10G (3.0%, 9/304) and E113D (2.6%, 8/304).

In LCR, a total of 41 nucleotide variations were detected in the 304 isolates. The most common LCR variations were G7193T, 7434CIns, G7521A, 7863ADel, which were found in 100% of HPV16 isolates. As shown in Table 3, a total of 13 novel nucleotide variations in LCR were found. A co-variation of 10 sites, including C7294T, G7319A, A7326G, T7350A, T7368G, T7503G, A7515C, G7553T, A7602G and A7740T in LCR was observed in 22 isolates (7.2%, 22/304).

Analyses by TFSEARCH software showed that in LCR, 25 (61.0%) of the 41 variations were located in the binding sites for transcription factors. The variation of G7193T resulted in loss of a putative binding site for the cellular transcription factor SRY. Furthermore, an insertion of 7434C resulted in a new binding site for the cellular transcription factor Oct-1, while a deletion of 7863A resulted in loss of a binding site for the cellular transcription factor E2. In the co-variation of 10 sites, the variation of A7515C resulted in loss of a binding site for the human activator AP-1, while the variation of A7602G resulted in an addition of binding site for the cellular transcription factor Oct-1.

### Association of HPV16 polymorphisms with persistent infection

Two lineages were found in the populations, including EUR lineage and As lineage. The association of HPV16 polymorphisms with persistent HPV infections was investigated by comparing the proportion of certain variant in women with persistent (n =168) and transient (n=106) HPV infections (Table 1). Among the populations infected by the HPV16 E variant of T350G, 16 women (9.5%, 16/168, 95% CI 14.8–29.9) were persistent infections and 12 women (11.3%, 12/106, 95% CI 15.5–36.5) were transient infections. The difference was not statistically significant (p=0.76). The As lineage being detected in 81 of the 168 (48.2%) persistently infected women and 22 of the 106 (20.8%) transiently infected women was associated with persistent infections (OR 0.3, CI 0.2-0.5).

### Association of HPV16 polymorphisms with risk for developing CIN2,3

The infection rate of women by HPV16 variants were compared between 137 women with <CIN2,3 (95 without CIN and 32 with CIN1, 10 with LSIL) and 167 women with ≥CIN2,3 (117 with CIN2,3 and 50 with cancer). Controlling for study, older women (OR 1.1, 95% CI 1.0–1.2) and those with a history of smoking (OR 1.0, 95% CI 0.5–2.0) were not at increased risk for developing ≥CIN2,3. The number of HPV genotypes per sample was similar between participants with <CIN2,3 (median of two genotypes) and those with ≥CIN-2,3 (median of three genotypes) (OR 1.1, 95% CI 0.9–1.4; p=0.34).

The As lineage was more frequent in pre-malignant and cervical cancer, and showed a statistically significant association for disease progression (OR 0.2, 95% CI, 0.1-0.3; P<0.001). While, the EUR lineage showed a statistically significant negative trend of association with the severity of >CIN2,3 (OR5.0, 95% CI, 2.9-8.3; P<0.001). Individual polymorphism that varies within lineages may make sense to look at differences between different disease groups. However, the 350T/G, the 94G/A polymorphism in the EUR lineage and the 131A/C polymorphism in the As lineage did not showed any particular association with comparing relative risk.

Two nonsynonymous variations of A131G (R10G) and T350G (L83V) were observed in HPV16 E6 gene. The variations of R10G/L83V occurred in 1 isolate from the 137 women with < CIN2,3 and 8 isolates from the 167 women with ≥CIN2,3. Controlling for study site, age and smoking, detection of the variant R10G/L83V (OR 4.7, 95% CI 1.0-23.5; *p*=0.04) was significantly associated with risk for developing ≥CIN2,3. The C7294T co-variation in LCR, which include 10 variations, occurred in 4 strains from the 137 women with <CIN2,3 and 18 strains from the 167 women with ≥CIN2,3, and was significantly associated with development of ≥CIN2,3 (OR 3.8, 95% CI 0.8-19.6; *p*=0.03) (Table 4). The association of cervical diseases with the other variations of transcription factor binding sites in LCR could not be conducted, because of the small number of the variations found in cases with ≥CIN2,3.

**Table 4:** Association between HPV16 E6 and LCR gene and CIN2/3 in 304 women

**No. of isolates with disease grade (%) n=304**				
**Variable**	**<CIN2,3 (n = 137)**	**≥ CIN2,3 (n =167)**	Crude OR (95% CI)*	Adjusted OR (95% CI) *
**Number of HPV types per sample**				
**No. of types (median)**	2	3	1.1 (0.9–1.4)	nd
**EUR lineage**	**113(82.5)**	**78(46.7)**	**5.0 (2.9-8.3)**	**5.8(3.2-9.4)**
E-350G/T	13(11.5)	17(21.8)	0.5(0.2-1.0)	nd
E-94G/A	17(15.0)	18(23.1)	0.6(0.3-1.2)	nd
**As lineage**	**24(17.5)**	**89(53.3)**	**0.2 (0.1-0.3)**	**0.3(0.2-0.8)**
As-131A/C	9(37.5)	23(25.9)	1.4(0.4-3.4)	nd
**E6 variations**				
Referent variants	84 (61.3)	88 (52.6)	1.0(reference)	1.0(reference)
R10G/ L83V	1(0.7)	8(4.8)	0.1(0.0-1.0)	0.1(0.1-0.9)
**LCR variations**				
Referent variants	137 (100)	167 (100)	1.0(reference)	1.0(reference)
Co-variation of C7294T^a^	4 (1.5)	18 (10.8)	0.2 (0.1-0.6)	5.7(0.1-0.7)

Note: nd: no data; *:<CIN2,3 vs >CIN2,3; a: Co-variation of C7294T include: C7294T, G7319A, A7326G, T7350A, T7368G, T7503G, A7515C, G7553T, A7602G and A7740T.

## Discussion

In the present study, it was examined whether intratype variations of E6 and LCR may affect persistent infection and have higher risk for developing cervical disease. Sequence variations in E6 gene and LCR were found in 304 HPV16 isolates from women with various grades of cervical lesions. Phylogenetic analysis of HPV16 variants with respect to the E6 gene and LCR was conducted to study the local distribution of different HPV16 lineages, according to the classification of Yamada et al. [14]. In our study, phylogenetic analysis of the E6 gene showed an extremely higher prevalence of the As lineage (49.7%), followed by E-prototype lineage (36.4%), E350G-lineage (9.9%), E131G-lineage (3.0%) and Af-1 variant (4.3%).

Different biological behaviors have been previously proposed for HPV16 infection. In Europe, it was reported that the frequency of Non-E variants, of the AA variant, in particular, increases strikingly with severity of lesion in infected women, suggesting that they could have a more relevant role in oncogenesis [17,18]. Although the sample population in a study by Tornesello et al. contains only one case infected with the AA variant, the result of histological examination revealed the presence of a carcinoma in situ [18]. Non-E variants have been demonstrated to associate with an increased risk of cervical intraepithelial lesion, as compared with E variants [10,19]. Additionally, a higher prevalence of AA variants was found in squamous cell carcinoma samples than in normal smears [20-22]. In the study, only two lineages were found in the population, including EUR lineage and As lineage. Accordance with previous researches, the association between As lineage infection and an increased risk of persistent infection was observed in our study. The As lineage was more frequent in pre-malignant and cervical cancer, and showed a statistically significant association for disease progression. While, the EUR lineage showed a statistically significant negative trend of association with the severity of >CIN2,3.

HPV16 E6 polymorphisms have been shown, although inconsistently, to be associated with CIN or cervical cancer [23,24]. As reported previously, HPV16 molecular variants did not evolve from random mutational events as variation sites and combinations of variations were shared by several isolates and variants [25,26]. In Europe, the variants harboring the HPV16 E6 gene substitution of T350G (L83V) seem to be associated with an increased risk of persistent infection and cytological progression to CIN 2, 3 and SCC [4,24]. However, this finding has not been confirmed in other European cohorts and in studies conducted in other parts of the world [22,27]. The biological significance of the L83V variation in HPV16 remains uncertain [24,28]. In our study, the alone L83V variation in HPV16 E6 was not associated with an increased risk of CIN2,3, but together with R10G, the nonsynonymous variations R10G/L83V were associated significantly with an increased risk for developing CIN2,3. It has been demonstrated that amino acid changes of R10G/L83V can alter the ability of E6 to bind and degrade p53, induce antiapoptotic signals or alter their binding affinity with host HLA [5].

HPV16 variants have been refined by the LCR, a variable non-coding segment of HPV genome [5]. Non-coding regions are less restricted in their ability to accumulate and tolerate variations [26]. Polymorphism of HPV16 LCR has been shown to confer a higher risk of developing persistent HPV infection, CIN2,3 or cancerous anogenital lesions[25,29]. A variation in the HPV33 LCR at nt 7732, resulting in loss of a putative binding site for USF, was found to be associated with CIN 2,3 [30]. As transcription of HPV oncogenes is under the control of promoter and enhancer elements in the LCR, variations within these motifs may alter the replication and transcription of HPV16 by abolishing, creating or modifying the affinity of a binding site for a cellular factor [31]. In our study, the C7294T co-variation in LCR was demonstrated to associate with risk for developing ≥CIN2,3. Because of the strong cosegregation of the co-variations of G7193T, 7434CIns, C7436T, G7521A, and 7863Adel, it is impossible to determine if each variation in it was individually responsible for risk for developing CIN2,3. It is predicted that the variation of A7515C in the C7294T co-variation can result in loss of a binding site for the human activator AP-1, while the variation of A7602G result in an addition of binding site for cellular transcription factor Oct-1. AP-1 is a Jun/Fos heterodimer complex protein that activates transcription of E6 and E7 genes. Mutations in the second binding site for AP-1 have been shown in vitro to reduce HPV31 transcription to a lesser extent than mutations in the first binding site [32]. Oct-1 might activate the epithelial- specific HPV16 enhancer by stabilizing the binding of a nuclear factor to the composite element, which in turn results in higher levels of enhancer activity[33]. The increased binding of Oct-1 to the LCR could result in increased transcriptional level of HPV16. The deletion at nt 7863A in HPV16 LCR will result in loss of a binding site for the cellular transcription factor E2. Mutations in the transcription factor E2 binding site have been shown in vitro to reduce HPV16 transcription [34]. Alternatively, variations in LCR may cosegregate with variations in HPV genes involved in replication, activation or transforming capacity of HPV16 genome that could be responsible for increased pathogenicity [13]. In vitro assessment, in which transcription factors can bind the LCR in any given transcriptional environment, is required to confirm the predicted effects of LCR variations.

## Conclusion

In summary, the finding of this study is that the R10G/L83V variations in E6 and C7294T co-variation in LCR was significantly associated with risk for developing ≥CIN2,3. Whether these associations are explained by a direct effect of E6 or LCR variations on HPV infection need to be complemented by functional studies further.

## Competing interests

The authors declare that they have no competing interests.

## Authors’ contribution

Zhengrong Sun carried out the molecular genetic studies, participated in the sequence alignment and drafted the manuscript. Zhitao Lu carried out the molecular genetic studies. Jianhua Liu participated in the sequence alignment. Guili Wang participated in performed the statistical analysis. Weiqiang Zhou, Lianxia Yang, Chao Liu, Bo Wang performed the clinical detection. Qiang Ruan conceived the study, and participated in its design and coordination and helped to draft the manuscript. All authors read and approved the final manuscript.

## Pre-publication history

The pre-publication history for this paper can be accessed here:

http://www.biomedcentral.com/1471-2407/13/459/prepub
